# 18F-FDG PET/CT Radiomics for Predicting Therapy Response in Primary Mediastinal B-Cell Lymphoma: A Bi-Centric Pilot Study

**DOI:** 10.3390/cancers17111827

**Published:** 2025-05-30

**Authors:** Fabiana Esposito, Luigi Manco, Luca Urso, Sara Adamantiadis, Giovanni Scribano, Lucrezia De Marchi, Adriano Venditti, Massimiliano Postorino, Nicoletta Urbano, Roberta Gafà, Antonio Cuneo, Agostino Chiaravalloti, Mirco Bartolomei, Luca Filippi

**Affiliations:** 1 Hematology, Department of Biomedicine and Prevention, University of Rome “Tor Vergata”, 00133 Rome, Italy; fabiana.e91@gmail.com (F.E.); lucrezia.demarchi@ptvonline.it (L.D.M.); adriano.venditti@uniroma2.it (A.V.); massimiliano.postorino@uniroma2.it (M.P.); 2 Medical Physics Unit, University Hospital of Ferrara, Via Aldo Moro 8, 44124 Ferrara, Italy; luigi.manco@ausl.fe.it (L.M.); giovanni.scribano@edu.unife.it (G.S.); 3 Department of Translational Medicine, University of Ferrara, 44124 Ferrara, Italy; sara.adamantiadis@unife.it (S.A.); roberta.gafa@unife.it (R.G.); 4 Nuclear Medicine Unit, Department of Onco-Hematology, Fondazione PTV Policlinico Tor Vergata University Hospital, 00133 Rome, Italy; nicoletta.urbano@ptvonline.it; 5 Hematology Unit, University of Ferrara, 44121 Ferrara, Italy; cut@unife.it; 6 Department of Biomedicine and Prevention, University of Rome “Tor Vergata”, 00133 Rome, Italy; agostino.chiaravalloti@uniroma2.it (A.C.); luca.filippi@uniroma2.it (L.F.); 7 Nuclear Medicine Unit, Onco-Hematology Department, University Hospital of Ferrara, 44124 Ferrara, Italy; m.bartolomei@ospfe.it

**Keywords:** PET/CT, 18F-FDG, radiomics, primitive mediastinal B-cell lymphoma, machine learning, artificial intelligence

## Abstract

Purpose: This bi-centric pilot study investigates the predictive value of pre-treatment [^18^F]FDG PET/CT radiomics for assessing therapy response in primary mediastinal B-cell lymphoma (PMBCL). Methods: All PMBCL patients underwent PET/CT with [^18^F]FDG between January 2011 and January 2022 at Policlinico Tor Vergata University Hospital of Rome (70% training and 30% internal validation cohort) and Sant’Anna University Hospital of Ferrara (external validation cohort). The Deauville score (DS) was used as a predictor of therapy response (DS1-DS3 vs. DS4/DS5). A total of 121 quantitative radiomics features (RFts) were extracted from manually segmented volumes of interest (VOIs) in PET and CT images, according to IBSI. ComBat harmonization was applied to correct the center variability of features, followed by class balancing with SMOTE. Two machine learning (ML) prediction models, the PET model and the CT model, were independently developed using robust RFts. For each ML model, two different algorithms were trained (i.e., Random Forest, RF, and Support Vector Machine, SVM) using 10-fold cross validation, tested on the internal/external validation set. Receiver operating characteristic (ROC) curves, area under the curve (AUC), classification accuracy (CA), precision (Prec), sensitivity (Sen), specificity (Spec), true positive (TP) scores, and true negative (TN) scores were computed. Results: The entire dataset was composed of 29 samples for the Rome cohort (23 from D1–D3 and 6 from D4/D5) and 9 samples for the Ferrara cohort (4 from D1–D3 and 5 from D4/D5). A total of 27 RFts were identified as robust for each imaging modality. Both the CT and PET models effectively predicted the Deauville score. The performance metrics of the best classifier (SVM) for the CT and PET models in external validation were AUC = 0.75/0.80, CA = 0.85/0.77, Prec = 0.97/0.67, Sen = 0.60/0.80, Spec = 0.98/0.75, TP = 75.0%/66.7%, and TN = 77.8%/85.7%, respectively. Conclusions: ML models trained on [^18^F]FDG PET/CT radiomic features in PMBLC patients could predict the Deauville score.

## Simple Summary

This bi-centric pilot study investigates the predictive value of pre-treatment [^18^F]FDG PET/CT radiomics for therapy response in primary mediastinal B-cell lymphoma (PMBCL). Patients from two Italian centers underwent baseline PET/CT, and the Deauville score (DS) was used to define response (DS1–3 vs. DS4–5). Radiomic features (RFts) were extracted from manually segmented PET and CT images and harmonized across centers. Two machine learning models (PET and CT) were trained with Random Forest and Support Vector Machine algorithms. In external validation, the best-performing SVM classifier showed AUCs of 0.80 (PET) and 0.75 (CT), with accuracies of 77% and 85%, respectively. Both models demonstrated strong specificity and precision. These results suggest that PET/CT radiomics-based ML models may assist in predicting treatment response in PMBCL patients.

## 1. Introduction

Primary mediastinal B-cell lymphoma (PMBCL) accounts for approximately 2–3% of all non-Hodgkin lymphomas (NHLs) and mostly occurs in young individuals (median age of 35) [1]. It is a distinct entity in the current WHO classification and is characterized by specific clinicopathological and molecular features, with a gene expression profile partially overlapping with nodular sclerosis classical Hodgkin lymphoma [2,3,4]. Most patients present with a bulky anterior–superior mediastinal mass, exhibiting compressive symptoms like dry cough, dyspnea, or chest tightness. Mediastinal syndrome with jugular vein distension, thoracic wall venous reticulation, and “cape” edema, associated with superior vena cava thrombosis/obstruction, may occur. The disease typically presents as localized (Ann Arbor stages I–II) [5,6].

Histologically, it presents as a diffuse infiltrate of medium-to-large cells with clear cytoplasm, prominent nucleoli, and marked fibrosis. Tumor cells express B-cell antigens (CD19, CD20, CD22, and CD79A), with 80% showing weak to moderate CD30 positivity [7,8]. First-line therapeutic approaches may include various anthracycline-based regimens, such as dose-adjusted EPOCH-R (etoposide, prednisone, vincristine, cyclophosphamide, doxorubicin, and rituximab), R-CHOP14 (rituximab, cyclophosphamide, doxorubicin, vincristine, and prednisone every 14 days), and R-MACOPB (rituximab, methotrexate, doxorubicin, cyclophoshamide, vincristine prednisone, and bleomycin), all of which have demonstrated efficacy with the exception of R-CHOP21 (rituximab, cyclophosphamide, doxorubicin, vincristine, and prednisone every 21 days), which appears less effective in terms of response and exhibits favorable response rates compared to other types of NHL [9,10,11,12,13,14]. Second-line chemotherapy yields poor outcomes in relapsed cases [8,15]. Consolidation radiotherapy following first-line treatment was previously considered the standard of care [3]. While this approach showed promising results, it was not without medium- and long-term side effects, including pulmonary and cardiac complications, as well as secondary malignancies [16,17,18]. Currently, the radiotherapy approach is evolving, with the recent literature showing favorable outcomes in a radiation-free strategy for patients who achieve complete remission after treatment [19].

[^18^F]Fluorodeoxyglucose ([^18^F]FDG) PET/CT, as in all aggressive lymphomas, is the gold standard for staging, interim evaluation, and assessment at the end-of-treatment (EoT). For treatment response, the guidelines recommend using the five-point Deauville score (DS) [20,21]. The IELSG-26 study reported a high negative predictive value of PET at the end of treatment but a low positive predictive value. Likewise, an initial study by Melani et al. suggested that a negative EoT [^18^F]FDG PET scan is a strong predictor of cure, whereas a single positive EoT FDG-PET scan does not reliably indicate treatment failure [22]. Furthermore, the same study [22] reported that serial follow-up [^18^F]FDG-PET imaging can effectively differentiate between residual disease and post-treatment inflammatory changes, aiding in the identification of patients who may benefit from additional radiotherapy. Nevertheless, the interpretation of EoT PET/CT can present diagnostic challenges, as the heightened metabolic activity observed in residual mediastinal masses may yield spurious positive results, thereby complicating the formulation of optimal clinical management strategies. The interpretation of a DS of 4 remains frequently challenging, often requiring a new biopsy of the residual mediastinal mass to differentiate between inflammatory processes and treatment non-response [23]. Nonetheless, a negative [^18^F]FDG PET/CT at the end of treatment remains a well-established prognostic marker of a favorable outcome in clinical practice [22].

Radiomics, an emerging field in medical imaging, offers a promising solution to this challenge by extracting quantitative features from PET/CT scans that reflect tumor heterogeneity, metabolic activity, and the spatial distribution of [^18^F]FDG uptake [24]. Unlike conventional imaging interpretation, which relies on visual assessment, radiomics enables a more detailed and objective analysis of tumor characteristics, potentially improving response assessment accuracy and reducing unnecessary interventions [25,26]. Given the relatively indolent nature of residual PMBCL masses and the risk of overtreatment, integrating radiomic biomarkers into clinical practice could help refine post-treatment strategies and minimize unnecessary radiotherapy in patients who have already achieved metabolic remission.

Despite its potential, research on the role of [^18^F]FDG PET-based radiomics in PMBCL remains limited, partly due to the relatively low incidence of this oncological condition [27,28]. The primary objective of this pilot study is to evaluate the predictive value of pre-treatment [^18^F]FDG PET/CT radiomics in assessing treatment response in PMBCL. Specifically, this study aims to determine whether radiomic analysis can differentiate between patients who achieve a complete metabolic response (DS 1–3) and those with persistent or progressive disease (DS 4/5) at the end of therapy.

## 2. Materials and Methods

### 2.1. Study Design

This retrospective, Italian, bi-centric study included newly diagnosed patients with PMBCL who were treated with rituximab (R) and anthracycline-based therapeutic regimens and underwent [^18^F]FDG PET/CT between January 2011 and January 2022 at Policlinico Tor Vergata University Hospital of Rome (training and internal validation cohort) and Sant’Anna University Hospital of Ferrara (external validation cohort).

The inclusion criteria were as follows: (a) age ≥ 18 years; (b) histologically confirmed diagnosis of PMBCL; (c) baseline [^18^F]FDG PET/CT performed prior to the initiation of systemic therapies; (d) availability of EoT PET/CT; (e) a follow-up period of at least 24 months; and (f) a complete medical history. Conversely, exclusion criteria included (a) concomitant diagnosis of other oncological conditions; (b) unavailable EoT PET/CT; and (c) incomplete medical history or a follow-up period of less than 24 months.

For each patient enrolled, the following clinical data were collected: gender, age at diagnosis, disease stage, presence of B symptoms, Eastern Cooperative Oncology Group (ECOG) performance status, lactate dehydrogenase (LDH) levels, International Prognostic Index (IPI) categorization, presence of extranodal involvement, type of R-based treatment, whether consolidative radiotherapy was administered, whether disease progression occurred, and patient status at the last follow-up (progressive, alive with no evidence of disease).

The endpoint of this study was to assess whether [^18^F]FDG PET/CT-based radiomics, evaluated on pre-treatment scans, could predict EoT PET/CT results. For this purpose, at the EoT, patients with Deauville scores of DS1–3 were classified as having achieved a complete metabolic response, while those with DS4/DS5 were categorized as having a partial metabolic response or progressive disease (D5—new lesions or progression of existing ones). Notably, in this study, the determination of relapse or progression was based on information obtained from follow-up data.

### 2.2. [^18^F]FDG PET/CT Imaging

All patients underwent PET/CT with [^18^F]FDG, which was carried out according to the current imaging guidelines [29]. Two distinct scanners were used: each participating center performed PET/CT acquisition and reconstruction according to a standardized protocol detailed in previously published papers [30,31] and reported in Supplementary Materials.

In each center, PET/CT image analysis was performed by two experienced nuclear medicine physicians using Advantage 4.7 software (GE HealthCare, Wisconsin, MI, USA) at Tor Vergata University Hospital and the Syngo.via workstation (Siemens Healthineers, Erlangen, Germany) at Sant’Anna University Hospital of Ferrara. Any lesion exhibiting tracer uptake higher than the background and not categorized as physiological was considered potentially pathological. For each patient, the sites and number of lesions with pathological uptake were documented. The primary mediastinal mass and, when applicable, pathological lymph nodes were identified on PET images. Circular regions of interest (ROIs) were manually drawn and automatically converted into 3D volumes of interest (VOIs) by the software, as shown in Figure S1. The most common semiquantitative parameters were collected, including maximum and mean Standardized Uptake Values (SUVmax and SUVmean, respectively), metabolic tumor volume (MTV), and total lesion glycolysis (TLG). In each center, VOI segmentation of the mediastinal mass and the most significant pathological lymph nodes was manually performed on [^18^F]FDG PET/CT images by two expert nuclear medicine physicians using a 40% SUVmax threshold, employing the aforementioned software.

### 2.3. Feature Extraction

Quantitative radiomics features (RFts) were extracted from each VOI in PET and CT images. The extraction process was performed separately using the Radiomics package and the 3D Slicer image computing platform, in accordance with IBSI standardization [32].

A total of 121 RFts were obtained from each segmented VOI in both imaging modalities. These features were classified into different categories: 14 RFts corresponded to the original image and mask, 14 to the Shape (3D) class, 18 to first-order intensity statistics, 24 to the Gray Level Co-Occurrence Matrix (GLCM), 16 to the Gray Level Run Length Matrix (GLRLM), 16 to the Gray Level Size Zone Matrix (GLSZM), 14 to the Gray Level Dependence Matrix (GLDM), and 5 to the Neighboring Gray Tone Difference Matrix (NGTDM).

### 2.4. Data Preprocessing and Balancing Strategy

In order to correct the center variability of features, which can result from differences in imaging protocols or equipment across different centers, ComBat harmonization was applied [33]. Subsequently, we employed the Synthetic Minority Oversampling Technique (SMOTE) to generate synthetic samples, aiming to achieve a more balanced class distribution [34].

The cohort from Policlinico Tor Vergata University Hospital was then split into 70% for model training and 30% for internal validation. External validation was performed using a dataset from Sant’Anna University Hospital. No additional normalization or transformations were applied to the data.

### 2.5. Statistical Analysis and Model Construction

A filter-based feature selection algorithm, implemented using a custom Python (Version 3.8) script, was used to identify robust and non-redundant RFts. To determine the most reproducible RFts, a significance threshold of *p* < 0.05 in the Wilcoxon–Mann–Whitney U test was applied.

Two machine learning (ML) prediction models, the PET model and the CT model, were independently developed. For each ML model, two different algorithms were trained: Random Forest (RF) and Support Vector Machine (SVM). The Orange data-mining platform was used for training, as well as for internal and external validation of both ML models based on PET and CT robust RFts.

The RF and SVM models were trained using 10-fold cross-validation (10FoldCV), tested on the internal validation set, and externally validated on the respective dataset.

Receiver operating characteristic (ROC) curves were plotted, and the area under the ROC curve (AUC), classification accuracy (CA), precision, sensitivity, specificity, true positive (TP) scores, and true negative (TN) scores were computed. These performance metrics were used to determine the best-performing algorithm for the PET and CT models.

## 3. Results

**Figure 1:**
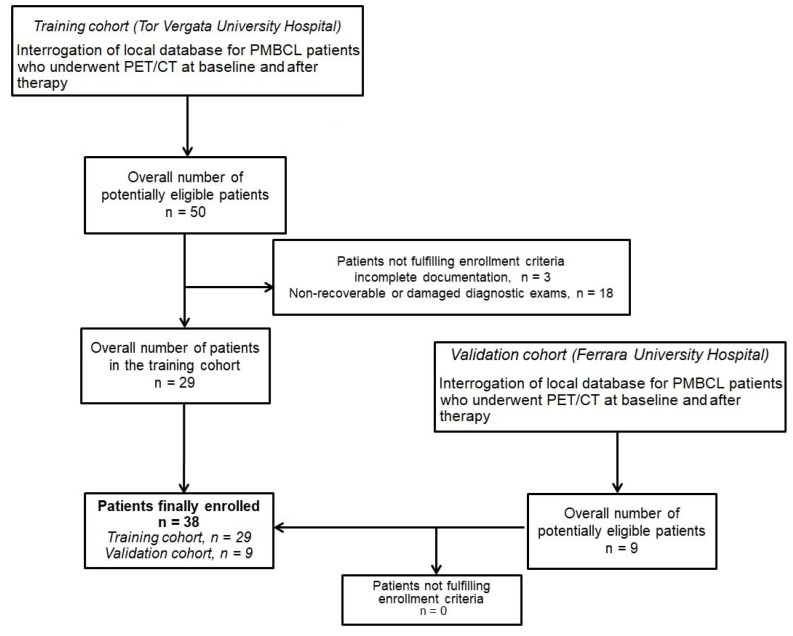
Flow-chart of patient selection. A total of 38 PMBCL patients undergoing PET/CT at baseline and after therapy were enrolled from two centers: 29 in the training cohort and 9 in the validation cohort.

Initially, 50 potentially eligible patients were identified in the training cohort. However, 21 patients were excluded due to incomplete documentation (*n* = 3) or non-recoverable/damaged diagnostic exams (*n* = 18), leaving 29 patients in the training cohort. In the validation cohort, nine patients were identified, all of whom met the inclusion criteria (Figure 1). Ultimately, 38 patients were enrolled in the study, with 29 in the training cohort and 9 in the validation cohort. The demographic and clinicopathological characteristics of the selected patients are summarized in Table 1.

### 3.1. Cohorts’ Composition, Characteristics, and Outcome

The median age was slightly higher in the validation cohort (39.5 years, range 25–58) than in the training cohort (35 years, range 18–58). The distribution of the IPI scores varied, with a greater proportion of patients in the training cohort presenting with lower IPI scores. Notably, B symptoms were absent in the validation cohort but present in nearly one-third of the training cohort. Most patients in both groups had a good performance status (ECOG 0–1). Bulky disease was frequent in both cohorts, while extranodal involvement was observed only in the training cohort. Regarding treatment, the training cohort received a variety of anthracycline-based regimens, predominantly R-MACOP-B, while all patients in the validation cohort were treated with RCHOP.

**Figure 2:**
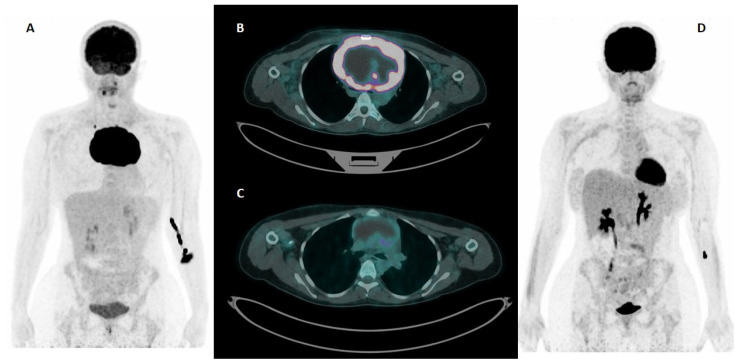
A case of PMBCL in a young woman (28 years old) from the University Hospital of Ferrara. Maximum intensity projection (**A**)—MIP—of baseline [^18^F]FDG PET/CT shows a large area of intense [^18^F]FDG uptake corresponding, in trans-axial fused PET/CT images (**B**), to a bulky mass of the anterior mediastinum. The image shows peripherical uptake with a “cold” core, corresponding to central necrosis. End-of-treatment [^18^F]FDG PET/CT shows a complete metabolic response (DS = 1) both in trans-axial fused PET/CT images (**C**) and at MIP (**D**). The patient did not experience disease relapse at follow-up (64 months).

At the end of treatment, the majority of patients in the training cohort (23 out of 29) achieved a DS of 1–3, whereas the validation cohort had a more evenly distributed response, with four patients classified as responders and five as non-responders (DS 4–5). A representative case of a patient with a large, hypermetabolic mediastinal mass at pre-treatment PET/CT, showing a complete metabolic response (DS1) after therapy, is illustrated in Figure 2. Consolidative radiotherapy was administered more frequently in the training cohort, and relapse occurred in only two patients. At the last follow-up, all patients in both cohorts were alive, with no evidence of disease, and the median follow-up time was 4.66 years in the training cohort and 3.64 years in the validation cohort.

### 3.2. Radiomic Analysis and Model Building

After applying the class balancing process using the SMOTE methodology, the entire dataset was composed of 47 samples for the training cohort (25 from DS1–DS3 and 22 from DS4/DS5) and 13 samples for the validation cohort (5 from DS1–DS3 and 8 from DS4/DS5). Among the 122 RFts extracted from CT and PET, 27 were identified as robust for each imaging modality. The full list of selected RFts is reported in Supplementary Material (Tables S1 and S2).

The performance of 10FoldCV across both learners demonstrated an AUC > 0.90 and a CA > 0.80 for the CT and PET models, respectively. During the training phase, the CT model reached the best performance, with the SVM classifier achieving an AUC > 0.97 and a CA > 0.88. In the PET model, the SVM classifier outperformed the RF classifier, reaching an AUC of 0.86 and a CA of 0.86. The performance of the two models, with both learners, during the training phase is shown in Table 2.

The internal validation, performed on the remaining 30% of the training dataset, yielded performance results consistent with those observed during training. In the CT model, the SVM classifier achieved an AUC of 0.90, with a TP of 80% and a TN of 75%.

The external validation, carried out on the validation cohort, maintained good classification capabilities for the models built. The set of performance scores for the validation processes are presented in Table 3.

**Figure 3:**
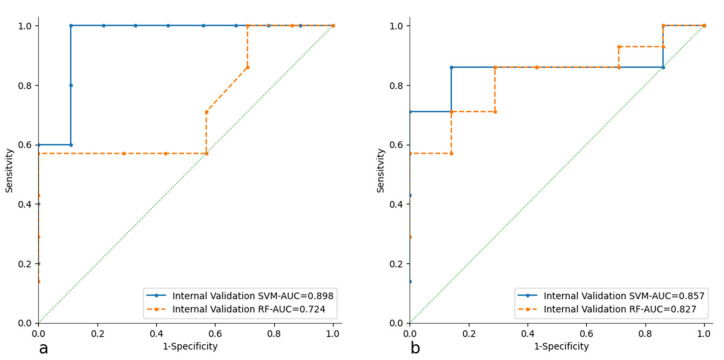
ROC curves of the CT (**a**) and PET (**b**) models with both learners (SVM and RF) in internal validation.

**Figure 4:**
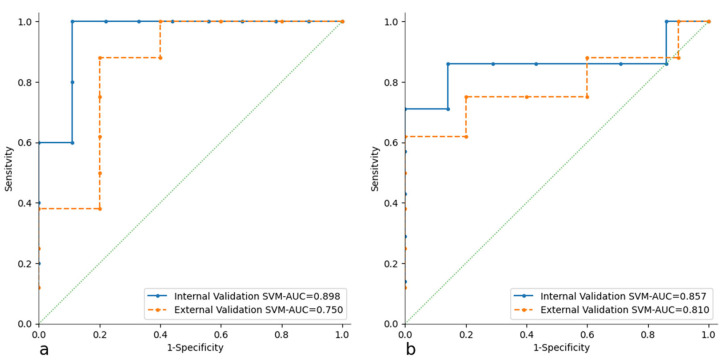
ROC curves of both internal and external validation of CT (**a**) and PET (**b**) models.

The ROC curves for the CT and PET models with both learners (SVM and RF) are shown in Figure 3. For the best-performing learner, identified as the SVM, the ROC curves for both internal and external validation are plotted (Figure 4).

Additionally, the ROC curves obtained during the training phase are provided in the Supplementary Materials (Figure S2).

**Figure 5:**
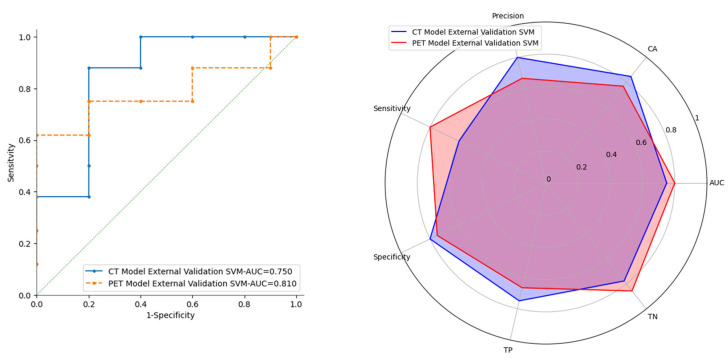
ROC curve comparison (**left**) and radial plot of the performance scores (**right**) of SVM learners for CT and PET models.

Finally, a comparison of the results was made, and a radial plot was created to visually compare the performance scores of SVM learners for each of the two ML models trained. Figure 5 presents a graphical comparison of the performance of the SVM, which emerged as the best learner in terms of performance across both models.

## 4. Discussion

[^18^F]FDG PET plays a crucial role in the management of PMBCL, offering metabolic insights that complement anatomical imaging. [^18^F]FDG PET/CT is not only essential for initial staging but also serves as a key tool in response evaluation, guiding subsequent therapeutic strategies. A significant challenge, especially after treatment, is the prevalence of false-positive results. Patients may present with a reduction in the size of the mediastinal mass, with residual focal findings evidenced at end of treatment [^18^F]FDG PET/CT, corresponding to a high DS (i.e., DS 4–5). However, in many cases, this does not reflect active disease, but rather necrotic inflammatory tissue due to chemotherapy, as confirmed by re-biopsy of the residual mediastinal mass. To date, there are no effective prognostic scores for PMBCL to understand the outcome of the disease at onset or drive therapeutic choices. The predictive value of [^18^F]FDG PET has been demonstrated in different studies; in particular, a negative PET scan at the end of chemoimmunotherapy is associated with prolonged progression-free survival (PFS) [35].

First-line treatment of PMBCL yields a high probability of cure. Regimens typically involve the use of rituximab in combination with intensive anthracycline-based chemotherapy. However, the prognosis is significantly impaired in patients who do not achieve a complete response or relapse after initial therapy [8,15]. Currently, also due to the rarity of the incidence of PMBCL, there is no standardized first-line treatment regimen, and there are no effective prospective randomized trials able to define the best chemoimmunotherapy treatment in this disease [9,36,37].

Initially, the standard treatment strategy included anthracycline-based chemotherapy with rituximab, as a CHOP-like regimen, followed by consolidation radiotherapy [38]. Dunleavy et al., in a prospective phase II study, demonstrated the efficacy of chemo-immunotherapy according to the DA-EPOCH-R regimen with omission of radiotherapy in patients with PMBCL [9]. Limited and conflicting data exist regarding the comparative effectiveness of different chemotherapy regimens. While some studies suggest DA-EPOCH-R superiority over R-CHOP, others report no significant outcome difference [37,39,40]. The DA-EPOCH-R regimen, while effective and potentially sparing patients from radiotherapy, presents a higher toxicity profile. This includes increased hospitalization, the need for central venous access (often difficult to find due to the presence of mediastinal bulky masses present at onset that expose patients to increased risk of venous thrombosis), and the increased use of granulocyte growth factors (G-CSFs) for more frequent and longer-lasting hematologic toxicities than the other regimens used in frontline PMBCL [36].

Recently, the IELSG37 study has revolutionized the therapeutic approach in patients, with PMBCL leading to the omission of radiotherapy in patients with complete metabolic response (DS1–3) [19,41]. A sub-analysis of this study compared the efficacy of different first-line chemo-immunotherapy regimens (R-CHOP 14, R-CHOP 21, R V/MACOP-B, DA-EPOCH-R), and, although DA-EPOCH-R has not demonstrated a statistically significant advantage, there is a trend towards efficacy in its favor compared to other therapeutic regimens [14]. It has to be emphasized that the choice of treatment often depends on the physician’s decision and does not reflect standard parameters. In order to identify high-risk patients, there are no prognostic scores to guide clinical practice in the treatment decision, and the IPI, used mainly in DLBCL, does not guide the choice of treatment and is not specific to PMBCL. In this context, it would be important to more effectively identify high-risk patients who might benefit from more intensive treatment, even facing an increased toxicity [38].

Given the role of [^18^F]FDG PET/CT in patients with PMBCL, recent studies have focused on the incorporation of quantitative functional parameters, such as MTV and TLG, to identify patients at high risk of treatment non-response who might benefit from more intensive treatments such as DA-EPOCH-R. A correlation was demonstrated between [^18^F]FDG PET-derived parameters, such as MTV and TLG and patient outcome in terms of OS and PFS [28,35,42]. One of the emerging findings in PMBCL is the role of baseline metabolic heterogeneity (MH) in predicting treatment outcomes. The presence of intratumoral heterogeneity, reflected in parameters such as textural features on PET scans, has been associated with an increased risk of treatment resistance and disease recurrence [42]. Tumors with high metabolic heterogeneity may harbor more aggressive clones, necessitating closer monitoring and potentially more intensive therapeutic approaches. However, the calculation of MH does not follow a standardized approach, which also leads to a certain variability in its clinical implication [43,44,45]. Advanced PET metrics may help mitigate these issues by offering more standardized cutoffs for residual disease assessment [46]. In this perspective, AI-driven PET analysis holds the potential to improve diagnostic accuracy by reducing interobserver variability and enabling automated risk stratification [47,48]. The integration of radiomic analysis into PET interpretation could refine prognostic assessments, enabling personalized treatment adjustments. Preliminary studies suggest that radiomic analysis can capture subtle variations in metabolic activity across voxels, reflecting fluctuations in tumor composition that are not immediately interpretable from a clinical standpoint [49]. However, these radiomic features have been found to strongly correlate with key biological tumor characteristics and have demonstrated significant prognostic value, being closely linked to treatment response and survival outcomes [48,50,51].

The most commonly used model building strategy involves splitting the training sample into two parts for training and testing, referred to as internal validation. The trained learners are the Random Forest (RF) and Support Vector Machine (SVM) algorithms. The performance of the two learners has no significant differences; both perform well, but the SVM demonstrates better adaptation to the data. This results in superior performance during 10FoldCV (AUC > 0.85, CA > 0.86, PRE > 0.85, SEN > 0.84, SPE > 0.85). This performance is replicated in both internal validation (AUC > 0.84, CA > 0.85, PRE > 0.85, SEN > 0.84, SPE > 0.86) and external validation (AUC > 0.75 CA > 0.77, PRE > 0.67, SEN > 0.60, SPE > 0.75). The evaluations of true positive (TP) and true negative (TN) scores are very interesting and significant, showing that there is a strong predictive power for the DS when analyzing the RFts of [^18^F]FDG PET/CT. In particular, the best scores are 77.80% for the TN with the CT model and 85.70% with the PET model. The statistically robust process of feature selection was confirmed by the stability of the two models. In fact, both the CT and PET models showed potential in predicting DS in PMBCL patients.

A common limitation often highlighted in radiomics studies is the lack of an external database, which prevents proper data validation. However, in our study, access to a database from another center allowed us to validate our models. Future developments of the project include validation on a larger cohort and prospective studies. In spite of its limited cohort of patients, our study offers elements that add value to the field, including a rigorous feature selection process, which effectively identifies the most representative radiomic features set for the binary problem under investigation. The use of harmonization techniques helps to mitigate the impact of different scanners, directly improving model performance in both the training and validation phases. As discussed by Orlhac and colleagues [52], harmonization in medical imaging can also be considered as a form of domain adaptation, where the goal is to produce images belonging to a single domain. It is important to highlight that the validation dataset, even if it is smaller than the training dataset, serves as a direct validation of the effective training of the proposed machine learning models. The use of an external unknown validation dataset in predictive ML models is essential to ensure reliability and to move closer to standardization in clinical practice. Finally, when comparing internal and external validation cohorts, serum LDH > 225 U/L was observed in all patients from the external validation cohort and in two-thirds of patients from the training cohort. Since increased LDH reflects greater and more aggressive neoplastic mass, we cannot exclude the possibility that the observed imbalance in baseline LDH—reflecting differences in tumor burden and aggressiveness between the training and external validation cohorts—contributed to the slightly lower true positive rates in the external set; however, our use of SMOTE to rebalance responders and non-responders likely mitigated this artifact. In this respect, the increasing sophistication of imaging techniques and the growing use of artificial intelligence and predictive models further highlight the need for a new standardization framework in multi- or bi-centric studies on PMBCL. This pilot study serves as a gateway to advanced clinical research in PET/CT and radiomics.

Radiomics, as observed in DLBCL and HL, may aid PMBCL risk stratification and future treatment decisions. Integrating biological, clinical, histological, and radiomic parameters could produce effective prognostic models for clinical use [40,53,54,55].

## 5. Conclusions

In conclusion, our pilot study suggests that radiomic features extracted from pre-treatment [^18^F]FDG PET/CT can effectively predict therapy response in PMBCL, as reflected by the DS. The application of machine learning models, particularly the SVM classifier, showed promising accuracy and robustness across both internal and external validations. Despite the limited sample size, these findings underscore the potential of integrating radiomics into clinical decision-making to guide personalized treatment strategies. Further large-scale, prospective studies are warranted to validate these preliminary results and refine prognostic models.

## Figures and Tables

**Table 1:** Characteristics of the study population in the training and validation cohorts.

Characteristics	Training Cohort (Rome) N (%)	Validation Cohort (Ferrara) N (%)
Patients	29	9
Male sex	9 (31%)	4 (44.4%)
Median age years (range)	35(18–58)	39.5(25–58)
IPI score		
1	11 (37.9%)	7 (77.8%)
2	10 (34.5%)	1 (0.1%)
3	3 (10.3%)	1 (0.1%)
4	1 (3.4%)	0 (0%)
>4	0 (0%)	0 (0%)
B symptoms	9 (31%)	0 (0%)
LDH		
≤225 U/L	19 (65.5%)	0 (0%)
>225 U/L	10 (34.5%)	9 (100%)
ECOG		
0–1	19 (65.5%)	9 (100%)
≥2	10 (34.5%)	0 (0%)
Bulky presentation	25 (86.2%)	8 (88.9%)
Extranodal localization	3 (10.3%)	0 (0%)
R-based regimen		
R-CHOP	4 (13.8%)	9 (100%)
R-MACOP-B	22 (75.9%)	0 (0%)
R-MACOP-B + autoTMO	2 (6.9%)	0 (0%)
R-MACOP-B + surgery	1 (3.4%)	0 (0%)
EoT PET response		
Responders (DS 1–3)	23 (79.3%)	4 (44.4%)
Not-responders (DS 4–5)	6 (20.7%)	5 (55.6%)
Consolidative RT		
Yes	18 (62.1%)	8 (88.9%)
No	11 (37.9%)	1 (11.1%)
Relapse		
Yes	2 (6.9%)	0 (0%)
Not	27 (93.1%)	9 (100%)
Patient status at last follow-up		
Alive with NED	28 (96.6%)	9 (100%)
Progressive	1 (3.4%)	0 (0%)
Median follow-up (years)	4.66	3.64

CHOP = chemotherapy; DS = Deauville score; ECOG = Eastern Cooperative Oncology Group; EoT = end of treatment; LDH = lactate dehydrogenase; MACOP = methotrexate Adriamycin cyclophosphamide oncovin prednisone; NED = no evidence of disease; RT = radiation therapy.

**Table 2:** Performance scores in 10FoldCV for each ML model.

	Training 10FoldCV	AUC	CA	PRE	SEN	SPE	TP	TN
CT Model	RF	0.92	0.88	0.88	0.88	0.88	88.90%	86.70%
	SVM	0.97	0.88	0.88	0.88	0.88	84.60%	75.00%
PET Model	RF	0.83	0.71	0.73	0.71	0.71	77.30%	90.90%
	SVM	0.85	0.86	0.85	0.84	0.86	83.30%	61.90%

AUC = area under the curve; CA = classification accuracy; PRE = precision; RF = random forest; SEN = sensitivity; SPE = specificity; SVM = support vector machine; TP = true positive; TN = true negative.

**Table 3:** Performance scores in the validation step for each ML model.

	Internal Validation	AUC	CA	PRE	SEN	SPE	TP	TN
CT Model	RF	0.72	0.64	0.65	0.64	0.64	88.90%	66.70%
	SVM	0.90	0.93	0.94	0.93	0.93	84.60%	75.00%
PET Model	RF	0.83	0.71	0.73	0.71	0.71	77.30%	80.00%
	SVM	0.86	0.85	0.85	0.84	0.85	83.30%	85.70%
	**External Validation**	**AUC**	**CA**	**PRE**	**SEN**	**SPE**	**TP**	**TN**
CT Model	RF	0.85	0.69	0.60	0.60	0.75	60.00%	75.00%
	SVM	0.75	0.85	0.97	0.60	0.98	75.00%	77.80%
PET Model	RF	0.74	0.69	0.57	0.80	0.63	57.10%	83.30%
	SVM	0.81	0.77	0.67	0.80	0.75	66.70%	85.70%

AUC = area under the curve; CA = classification accuracy; PRE = precision; RF = random forest; SEN = sensitivity; SPE = specificity; SVM = support vector machine; TP = true positive; TN = true negative.

## Data Availability

Datasets generated and analyzed during the study are available upon reasonable request to the corresponding author.

## Supplementary Materials

The following supporting information can be downloaded at: https://www.mdpi.com/article/10.3390/cancers17111827/s1, Figure S1: 25-year-old man with newly diagnosed primary mediastinal B-cell lymphoma. (A) Whole-body PET shows an extensive area of increased tracer uptake in the mediastinum. Axial (B) and coronal (C) fused PET/CT images demonstrate lesion segmentation using a 40% SUVmax threshold (yellow contour); Figure S2: ROC curves obtained during the training phase for the PET and CT-based models; Table S1: CT model selected robust radiomic features; Table S2: PET model selected robust radiomic features.
