# Supplementary material for: 18F-FDG PET/CT Radiomics for Predicting Therapy Response in Primary Mediastinal B-Cell Lymphoma: A Bi-Centric Pilot Study
Source: Cancers (Basel). 2025 May 30;17(11):1827. doi: 10.3390/cancers17111827 (PMC12153670; doi:10.3390/cancers17111827)
Supplement: Supplementary file 1 [file cancers-17-01827-s001.zip › cancers-3633200-supplementary.pdf]

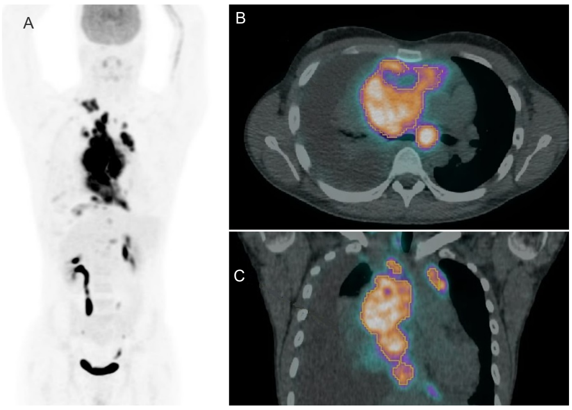

Figure S1: 25-year-old man with newly diagnosed primary mediastinal B-cell lymphoma. (A) Whole-body PET shows an extensive area of increased tracer uptake in the mediastinum. Axial (B) and coronal (C) fused PET/CT images demonstrate lesion segmentation using a 40% SUVmax threshold (yellow contour).

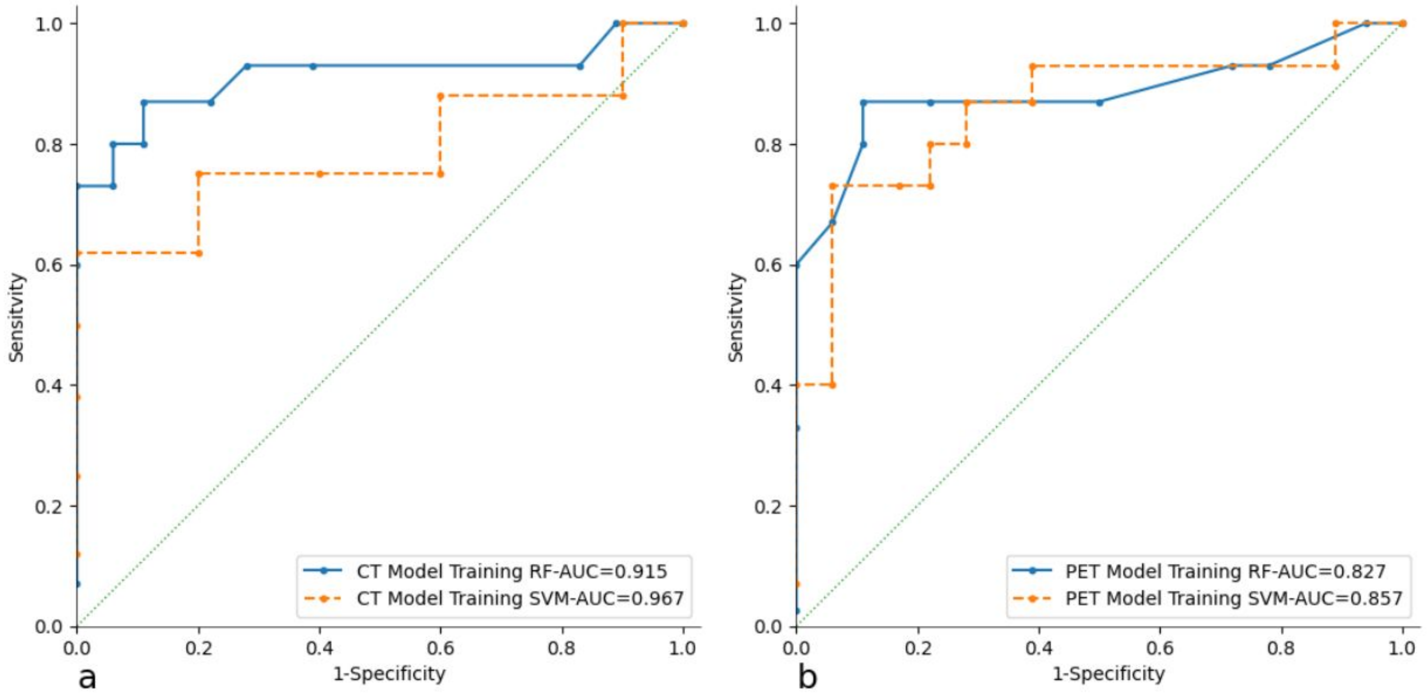

Figure S2 ROC curves obtained during the training phase for the PET and CT-based models, respectively

Table S1: CT Model selected robust radiomic features

Filter	Class	Radiomic feature	DS 0	DS1	tot	p
diagnostics	original	Maximum	3.06E+4±3.1E+2	3.04E+4±4.52E+2	3.05E+4±3.89E+2	1.36E-04
diagnostics	original	Mean	-9.41E+15±4.67E+15	-8.56E+15±3.16E+15	-9.01E+15±4.01E+15	7.96E-03
diagnostics	original	Minimum	-2.62E+4±4.83E+3	-2.27E+4±3.39E+3	-2.46E+4±4.52E+3	5.79E-03
diagnostics	original	VolumeNum	3.22E+0±4.4E+0	6.16E+0±5.76E+0	4.59E+0±5.24E+0	1.46E-02
diagnostics	original	VoxelNum	7.55E+4±6.42E+4	1.35E+5±7.11E+4	1.03E+5±7.31E+4	1.17E-03
original	firstorder	10Percentile	-1.18E+2±7.96E+2	1.03E+2±7.71E+1	-1.46E+1±5.88E+2	1.53E-02
original	firstorder	Energy	5.49E+9±6.39E+9	5.31E+9±4.62E+9	5.41E+9±5.57E+9	3.26E-02
original	firstorder	Entropy	1.41E+16±9.05E+15	7.56E+15±5.45E+15	1.11E+16±8.2E+15	4.64E-03
original	firstorder	Median	3.73E+2±5.54E+1	3.61E+2±3.97E+1	3.67E+2±4.85E+1	2.30E-02
original	firstorder	Minimum	-7.59E+3±2.56E+3	-8.22E+3±1.3E+3	-7.88E+3±2.07E+3	3.39E-02
original	firstorder	RootMeanSquared	1.29E+16±1.61E+16	9.6E+15±1.38E+16	1.14E+16±1.5E+16	1.70E-02
original	glcm	Correlation	7.17E+15±6.68E+15	4.41E+15±1.8E+15	5.88E+15±5.17E+15	1.17E-03
original	glcm	DifferenceVariance	1.11E+16±1.14E+16	1.8E+16±1.1E+16	1.44E+16±1.16E+16	3.77E-04
original	glcm	Idmn	9.57E+15±1.84E+15	7.9E+15±2.51E+15	8.79E+15±2.31E+15	1.03E-02
original	glcm	JointEnergy	1.37E+16±8.24E+15	1.9E+16±5.71E+15	1.62E+16±7.58E+15	1.44E-02
original	glcm	JointEntropy	2.38E+16±1.33E+16	2.48E+16±1.07E+16	2.43E+16±1.2E+16	1.44E-02
original	glcm	SumSquares	1.37E+16±1.07E+16	7.19E+15±5.41E+15	1.07E+16±9.15E+15	3.78E-02
original	gldm	SmallDependenceHighGrayLevelEmphasis	9.47E+15±1.14E+16	1.86E+16±1.02E+16	1.38E+16±1.17E+16	1.11E-04
original	glrlm	LongRunEmphasis	9.81E+15±1.1E+16	1.03E+16±7.95E+15	1.01E+16±9.59E+15	3.18E-03
original	glrlm	RunLengthNonUniformity	1.09E+16±1.28E+16	1.97E+16±1.67E+16	1.5E+16±1.53E+16	1.91E-02
original	glszm	LargeAreaHighGrayLevelEmphasis	1.61E+16±1.42E+16	2.38E+16±1.07E+16	1.97E+16±1.32E+16	1.58E-04
original	glszm	ZonePercentage	1.88E+16±1.62E+16	1.81E+16±1.16E+16	1.85E+16±1.41E+16	3.15E-02
original	ngtdm	Busyness	1.22E+16±1.08E+16	8.28E+15±2.9E+15	1.04E+16±8.29E+15	2.38E-03
original	ngtdm	Coarseness	5.8E+15±6.68E+15	3.1E+15±1.66E+15	4.54E+15±5.14E+15	2.15E-02
original	shape	Elongation	9.03E+15±6.94E+15	1.5E+16±1.23E+16	1.18E+16±1.01E+16	1.17E-02
original	shape	Maximum2DDiameterSlice	9.76E+15±9.31E+15	1.14E+16±3.E+15	1.05E+16±7.07E+15	5.57E-03
original	shape	SurfaceVolumeRatio	1.3E+16±9.36E+15	5.83E+15±3.39E+15	9.65E+15±8.E+15	1.03E-02

DS= Deauville Score; GLCM=Gray-Level Co-occurrence Matrix; GLDM= Gray Level Dependence Matrix ; GLRLM=Gray-Level Run Length Matrix; GLSZM=Gray-Level Size Zone Matrix; NGTDM=Neighboring Gray-Tone Difference Matrix.

Table S2: PET Model selected robust radiomic features

Filter	Class	Radiomic feature	DS 0	DS1	tot	p
original	firstorder	90Percentile	9.53E+15±6.55E+15	1.28E+16±7.53E+15	1.11E+16±7.14E+15	3.92E-02
original	firstorder	InterquartileRange	7.19E+15±8.58E+15	1.14E+16±1.19E+16	9.14E+15±1.04E+16	4.06E-02
original	firstorder	Maximum	1.21E+16±1.04E+16	2.09E+16±8.12E+15	1.62E+16±1.03E+16	1.78E-04
original	firstorder	Skewness	7.85E+15±8.81E+15	1.23E+16±6.54E+15	9.92E+15±8.06E+15	3.03E-02
original	firstorder	Variance	8.41E+15±7.11E+15	3.39E+15±3.4E+15	6.06E+15±6.17E+15	9.21E-05
original	glcm	ClusterProminence	2.93E+15±6.93E+15	9.12E+15±8.34E+15	5.83E+15±8.16E+15	3.24E-02
original	glcm	ClusterShade	4.06E+15±5.68E+15	8.01E+15±4.68E+15	5.91E+15±5.55E+15	3.91E-02
original	glcm	ClusterTendency	2.72E+15±6.42E+15	9.12E+14±4.52E+15	1.87E+15±5.63E+15	8.07E-05
original	glcm	DifferenceVariance	5.98E+15±1.16E+16	1.39E+16±1.47E+16	9.68E+15±1.36E+16	1.12E-02
original	glcm	Imc2	4.26E+15±6.13E+15	1.14E+16±7.47E+15	7.62E+15±7.63E+15	1.64E-02
original	glcm	JointEnergy	2.5E+15±5.02E+15	7.06E+15±7.27E+15	4.63E+15±6.52E+15	3.24E-02
original	glcm	MCC	2.85E+15±5.08E+15	7.44E+15±8.19E+15	5.E+15±7.03E+15	2.57E-02
original	glcm	SumSquares	2.05E+15±4.3E+15	4.24E+15±3.92E+15	3.08E+15±4.23E+15	2.67E-02
original	gldm	DependenceNonUniformity	1.45E+16±9.9E+15	1.68E+16±8.85E+15	1.55E+16±9.4E+15	1.56E-02
original	gldm	DependenceNonUniformityNormalized	1.67E+16±1.76E+16	2.02E+16±1.61E+16	1.83E+16±1.68E+16	1.12E-02
original	gldm	LargeDependenceEmphasis	1.06E+16±1.09E+16	1.56E+16±7.59E+15	1.29E+16±9.76E+15	1.70E-02
original	gldm	SmallDependenceHighGrayLevelEmphasis	1.2E+16±9.8E+15	2.08E+16±8.16E+15	1.62E+16±1.E+16	5.83E-03
original	glrlm	GrayLevelNonUniformity	1.58E+16±1.03E+16	1.94E+16±9.39E+15	1.75E+16±9.94E+15	3.64E-02
original	glrlm	LongRunLowGrayLevelEmphasis	1.2E+16±7.23E+15	1.71E+16±9.15E+15	1.44E+16±8.49E+15	1.12E-02
original	glrlm	RunEntropy	1.44E+16±1.47E+16	2.06E+16±1.4E+16	1.73E+16±1.46E+16	2.50E-03
original	glrlm	ShortRunEmphasis	1.65E+16±1.4E+16	9.61E+15±5.72E+15	1.33E+16±1.13E+16	2.32E-02
original	glszm	LargeAreaEmphasis	3.34E+15±8.11E+15	5.68E+15±1.11E+16	4.44E+15±9.58E+15	4.36E-02
original	glszm	LargeAreaHighGrayLevelEmphasis	2.68E+15±6.68E+15	1.87E+14±1.47E+15	1.51E+15±5.09E+15	7.96E-03
original	glszm	LargeAreaLowGrayLevelEmphasis	3.22E+15±8.69E+15	6.16E+15±1.24E+16	4.6E+15±1.06E+16	3.15E-02
original	glszm	SmallAreaEmphasis	4.16E+15±6.18E+15	1.12E+16±8.25E+15	7.44E+15±7.97E+15	2.07E-02
original	ngtdm	Complexity	5.29E+15±1.05E+16	1.45E+16±1.43E+16	9.58E+15±1.31E+16	3.69E-03
original	ngtdm	Strength	2.62E+15±4.54E+15	8.83E+15±7.63E+15	5.53E+15±6.86E+15	1.17E-02

DS= Deauville Score; GLCM=Gray-Level Co-occurrence Matrix; GLDM= Gray Level Dependence Matrix; GLRLM=Gray-Level Run Length Matrix; GLSZM=Gray-Level Size Zone Matrix; NGTDM=Neighboring Gray-Tone Difference Matrix.
